# Clinical, Dermoscopic, and Histological Characteristics of Melanoma Patients According to the Age Groups: A Retrospective Observational Study

**DOI:** 10.3390/life13061369

**Published:** 2023-06-12

**Authors:** Monika Słowińska, Iwona Czarnecka, Robert Czarnecki, Paulina Tatara, Anna Nasierowska-Guttmejer, Małgorzata Lorent, Szczepan Cierniak, Witold Owczarek

**Affiliations:** 1 Department of Dermatology, Central Clinical Hospital Ministry of Defense, Military Institute of Medicine—National Research Institute, Szaserow 128, 04-141 Warsaw, Poland; iczarnecka@wim.mil.pl (I.C.); ptatara@wim.mil.pl (P.T.); wowczarek@wim.mil.pl (W.O.); 2 Evimed Medical Centre Ltd., Private Dermatologic Practice, JP Woronicza 16, 02-625 Warsaw, Poland; 3 Department of Cardiology, LUX MED Oncology, Limited Liability Company, St. Elizabeth Hospital, Goszczynskiego 1, 02-616 Warsaw, Poland; robert.czarnecki@luxmed.pl; 4 Department of Pathomorphology, Central Clinical Hospital of Ministry of Interior and Administration—National Medical Institute, Woloska 137, 02-507 Warsaw, Poland; anna.nasierowska@cskmswia.pl; 5 Faculty of Medicine, Lazarski University, Swieradowska 43, 02-662 Warsaw, Poland; 6 Department of Pathomorphology, Central Clinical Hospital Ministry of Defense, Military Institute of Medicine—National Research Institute, Szaserow 128, 04-141 Warsaw, Poland; mlorent@wim.mil.pl (M.L.); scierniak@wim.mil.pl (S.C.)

**Keywords:** melanoma, dermoscopy, nevi, age factors, comorbidity

## Abstract

Background: Although the role of melanoma risk factors is well documented, their correlation with patients’ age is less frequently analyzed. Method: The analysis was performed among 189 melanoma patients in different age groups, including <30 years, 31–60 years, and >60 years, to investigate the risk factors, topography, and coexistence of morphological features of 209 melanomas (dermoscopic and histopathological). Results: Among the youngest age group, no correlation with the presence of estimated risk factors was found. The most common dermoscopic pattern was spitzoid and multicomponent asymmetric. The group of middle-aged patients was the most diverse in terms of the occurrence of risk factors, solar lentiginosis, dermoscopic patterns, topography, histological subtypes, and invasiveness of melanomas. The oldest group characterized a strong correlation between solar lentiginosis, NMSC comorbidity, the prevalence of facial melanomas, the dermoscopic pattern of melanoma arising on chronic sun-damaged skin, and regression. Conclusion: The findings regarding the presence of age-specific features in melanoma patients, especially in the youngest and middle-aged groups, might be helpful for clinicians and to target secondary prevention efforts.

## 1. Introduction

Over the past few decades, there has been a large increase in the incidence of melanoma and non-melanoma skin cancers (NMSC), especially among the white population [1,2]. A worldwide total of 325,000 new melanoma cases and 57,000 melanoma-related deaths were estimated for 2020 with wide geographic variations [1]. With the continuation of the current rates, it is estimated that there will be a 50% increase in melanoma incidence and a 68% increase in melanoma deaths by the year 2040 [1]. Since reducing melanoma mortality is an unmet need, secondary prevention can be reconsidered to increase the effectiveness of the early detection of patients in a situation of limited health care capacity.

Numerous association studies have confirmed the correlation of known patient-related risk factors, multiple genetic factors, and lifestyle or iatrogenic factors [3,4,5,6,7]. Estimates of their effects indicate that high intermittent or intentional UV exposure increases the risk by about 60%; indoor tanning increases the risk by about 20%; a history of sunburns doubles the risk; similarly to skin prototype I, skin prototype II increases the risk by 80%; multiple acquired (common) nevi increase the risk sevenfold; and having at least five atypical nevi increases the risk sixfold [4]. Among patients undergoing dermoscopic screening, the above-mentioned risk factors occur with different frequencies, but their higher co-occurrence should be expected with age. 

Independently, epidemiological data indicate an increase in the incidence of melanoma in all age groups. Based on epidemiological data from the National Cancer Institute and the SEER Registry regarding the cutaneous melanoma incidence rates by age at diagnosis within the years 2016 and 2020 (Supplement S1) [8] and from the Polish National Cancer Registry regarding the crude incidence rates in the period 2016–2020 for the adult population (Supplement S2) [9], two increases in incidence were found—for the population over 30 years of age and for the population over 60 years of age. Therefore, the aim of the study was to analyze adult melanoma patients in three age groups in terms of the coexistence of morphological features of melanomas (dermoscopic and histological) and patient-related risk factors that manifested clinically.

## 2. Materials and Methods

Retrospective analysis included consecutive adult patients referred to a dermoscopic skin examination at Dermatology Department, Central Clinical Hospital Ministry of Defense, Military Institute of Medicine—National Research Institute or private dermatological practice (Evimed Medical Centre Ltd., Warsaw, Poland) in Warsaw between 1 January 2015 and 31 October 2022. The study protocol was approved by the Local Ethics Committee (#21/WIM/2021, 19 May 2021).

Patients’ medical records were evaluated based on the following:

- Epidemiologic data (gender, age at the moment of melanoma diagnosis, and location of melanoma), family or personal history of melanoma, and previous non-melanoma skin cancer (NMSC).
- The topography of melanoma was described as head and neck, trunk, upper limb and lower limb (including acral melanomas), and special locations (nail apparatus, mucous membrane of the oral or genital area, and eye).
- Melanoma risk factors that manifested clinically, including multiple acquired melanocytic nevi (above 50 melanocytic nevi), atypical nevus syndrome, skin phototype I or II, solar lentiginosis (as a marker for sun burn episodes), previous/coexisting non-melanoma skin cancer (NMSC), and genetic syndromes.
- Presence of histopathological report of melanoma. The histopathological subtypes of melanoma were evaluated as lentigo maligna (facial and extrafacial), lentigo maligna melanoma (facial), superficial spreading melanoma, nodular melanoma, spitzoid melanoma, nevoid melanoma, and desmoplastic melanoma. The melanoma invasiveness was described according to the TNM staging system/8th AJCC classification as pTis, pT1, pT2, pT3, and pT4 [10].
- Videodermoscopic documentation of melanoma. The dermoscopic pattern of melanoma was allocated to one of the following subtypes: multicomponent asymmetric, spitzoid, melanoma on sun damaged skin, hypomelanotic/amelanotic, homogenous, nodular, melanoma on face, and melanoma in special location (nail apparatus/acral/mucous membranes). The dermoscopic regression structures were regarded as present or absent.

The dermoscopic documentation was performed with the use of Fotofinder HD 800 or Medicam 1000 (FotoFinder Systems GmbH, Bad Birnbach, Germany) or Mole Max (Derma Medical Systems Handels u. Entwicklungs GmbH Vienna, Austria) and captured in polarized light and at the same 20-fold magnification.

Patients were enrolled in the study based on the inclusion and exclusion criteria listed below. Inclusion criteria: at least 18 years old at time of melanoma diagnosis; the full pathological report documented primary melanoma diagnosis according to the TNM staging system/8th AJCC classification including topography and melanoma subtype; pathological reports documented NMSC diagnosis; full videodermoscopic documentation enabling the classification of a dermoscopic pattern of melanoma; presence of melanocytic nevi; concomitant NMSC; solar lentiginosis; a skin phototype; information about the melanoma diagnosis among close relatives present in patient’s medical chart. Exclusion criteria: a lack of videodermoscopic documentation; lack of pathological report of primary melanoma; recurrent melanoma; melanoma of unknown primary location; metastatic melanoma after the excision of the primary lesion; melanoma in a location that prevents the dermoscopic/videodermoscopic examination; lack of data enabling the assessment of melanoma risk factors.

### Statistical Analysis

Statistical analysis was performed using R software environment (version 4.2.2 “Innocent and Trusting”; R Foundation for Statistical Computing, Vienna, Austria); https://www.R-project.org accessed on 7 November 2022; RStudio (Integrated Development Environment for R, version 2022.07.2+567 “Spotted Wakerobin” Release; Boston, MA, USA); http://www.rstudio.com accessed on 7 November 2022; and R packages including tidyverse, knitr, summarytools, kableExtra, crosstables, broom, scales, FSA, and epitools [11]. 

Frequencies of count data were calculated with cross tables. Differences in frequencies were estimated with Fisher’s exact test (expected counts in some groups were lower than 5). Differences in means of continuous numerical data were calculated with Kruskal–Wallis rank test (numerical data in whole data and groups were not normally distributed). Differences in means between multiple groups were calculated with Dunn’s test with Bonferroni’s *p*-value correction. Crude odds ratios with 95% confidence intervals for pairs of binomial variables in age groups were calculated using conditional maximum likelihood estimation. Odds ratios adjusted for age and gender with 95% confidence intervals were calculated using multivariable logistic regression.

## 3. Results

The data from the dermoscopic visits of 7367 patients (aged 2 months–92 years) admitted in the period 1 January 2015–31 October 2022 to two dermatological sites were analyzed, identifying 269 melanomas in 248 patients (aged 20–90 years; median 45 years; mean 49.3 years). As the videodermoscopic documentation of 209 melanomas was available in 189 patients (63.5% female and 36.5% men), this group was further analyzed. The summary of the epidemiological, clinicopathological. and dermoscopic data are presented in Table 1.

### 3.1. The Characteristics of the Melanoma Patients

Among the 189 patients (aged 20–90 years) enrolled in the study, the mean age at the diagnosis of melanoma was 51.6, and the median age was 47 years. Among 209 melanomas 61.7% (129/209) were detected during the first dermoscopic visit, and 38.3% (80/209) were detected during the monitoring visits. There was no statistical difference in the invasiveness of melanomas, although most small–size melanomas were detected during the monitoring visits as new lesions. Eighteen patients (9.5%) were diagnosed with at least 2 melanomas, and one (0.5%) was diagnosed with 3 melanomas. Among the 18 (8.6%) patients, melanoma had occurred in an immediate family member. 

The melanoma risk factors manifested clinically by atypical nevus syndrome or by numerous acquired nevi (ANS/NAN) were found in 121 (64%) patients, solar lentiginosis was found in 87 (41.6%) patients, and 41 (21.7%) patients had previous or coexisting NMSCs (nearly all had basal cell carcinoma—BCC). No genetic syndromes were detected among the patients. The group of 189 patients was very homogeneous in terms of skin phototype, as only 6 (3.2%) patients had phototype I or III, while the remaining 177 (93.6%) patients had phototype II. In 29 cases, melanoma occurred in a special location: 22 (10.5%) on the face, 3 (1.4%) on the scalp, 4 (1.9%) acral, 2 (1%) on mucous membranes (oral mucosa and vulva), and 1 (0.5%) within the nail apparatus. 

The majority of melanomas (84.7%) were diagnosed as thin melanoma (lentigo maligna, melanoma in situ, or pT1 stage), and the superficial spreading histological type was predominant (77%). Nodular melanoma (NM) was rarely detected and consisted of 5.7% of cases. Spitzoid, desmoplastic, or nevoid melanomas were not found in pathological reports. The predominant dermoscopic melanoma pattern was multicomponent asymmetric, with 91 patients (43.5%); followed by spitzoid, with 37 patients (17.7%); melanoma on chronically sun damaged skin, with 25 patients (12%); and melanoma on the face, with 22 patients (10.5%). 

### 3.2. The Analysis between the Individual Age Groups

**Figure 1:**
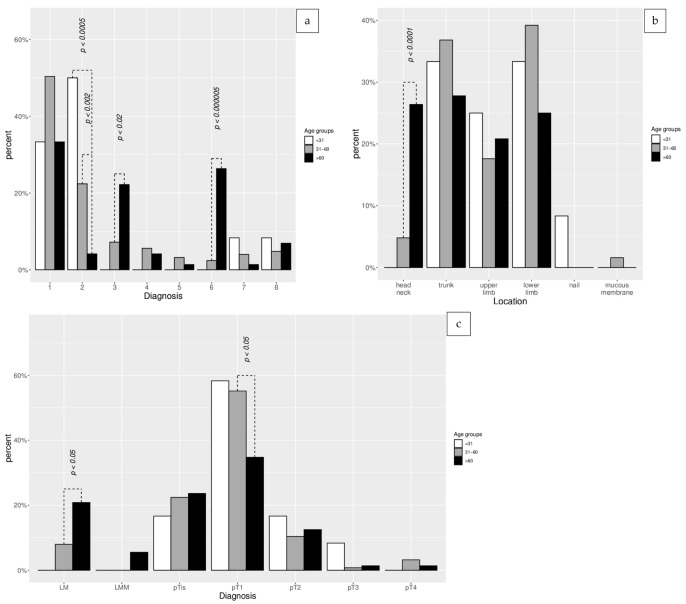
The Fisher’s exact tests for count data with simulated *p*-value analysis regarding the differences in melanoma location (**a**). The histopathological diagnosis (**b**) and the dermoscopic melanoma patterns (**c**) between the individual age groups (below 31 years, 31–60 years, and over 60 years). Legend for dermoscopic patterns on axis (x) for Figure 1c: 1—multicomponent asymmetric; 2—spitzoid; 3—melanoma on sun damaged skin; 4—hypomelanotic/amelanotic; 5—homogenous; 6—melanoma on face; 7—melanoma in special location (nail/acral/mucous membranes); 8—nodular.

The analysis between the individual age groups revealed statistically significant differences in the frequencies of multiple melanocytic nevi (*p* < 0.0005), previous/concomitant NMSC (*p* < 0.0005), solar lentiginosis (*p* < 0.0005), melanoma location (*p* < 0.001), melanoma histopathology (*p* < 0.01), melanoma dermoscopic pattern (*p* < 0.0005), and regression structures (*p* < 0.001) (Table 1). Figure 1 presents detailed data regarding the statistically significant differences among the individual age groups with regard to melanoma location, histopathological diagnosis, and the dermoscopic melanoma pattern. 

The group of patients younger than 31 years old had low statistical power due to the small number of melanomas (12 cases). In comparison to other age groups, striking differences were observed for the dermoscopic pattern of melanoma. The most common melanomas were spitzoid (16.2% of all/50% in the age group) and multicomponent asymmetric (4.4% of all/33.3% in the age group), and the age group also had the lowest incidence of regression structures (1.6% of all/8.3% in the age group) and solar lentiginosis (2.3% of all/16.7% in the age group). No cases of previous/coexisting NMSCs, melanoma located on the head/neck, and mucous membrane area were detected in the group. 

The group of patients aged 31–60 years was characterized by the presence of personal or familial melanoma incidence (74.3% of all/20.8% in the age group) and a high number of melanocytic nevi (70.3% of all/77.6% in the age group). Another feature was the wide diversity of dermoscopic melanoma patterns. The most common dermoscopic patterns were multicomponent asymmetric (69.2% of all/50.4% in the age group), spitzoid (75.7% of all/22.4% in the age group), hypomelanotic/amelanotic (70% of all/5.6% in the age group), and homogenous (80% of all/3.2% in the age group). The group of patients aged 31–60 years was also characterized by a higher incidence of thick melanomas (pT2—54.1% of total/10.4% in the age group; pT3—33.3% of all/0.8% in the age group; pT4—80% of all/3.2% in the age group). The topography of melanomas revealed the predominance of melanoma location on the lower limbs (69% of all/39.2% in the age group) and a general high diversity of melanoma locations, including extrafacial melanomas in special areas (66.6% of all/1.6% in the age group) (Figure 1).

The oldest group of melanoma patients is characterized by the common presence of solar lentiginosis (60.9% of all/73.6% in the age group), the highest incidence of facial melanoma (86.3% of all/26.4% in the age group), and lentigo maligna (60% of all/20.8% in the age group). The predominant dermoscopic melanoma patterns were melanoma on the face (86.3% of all/26.4% in the age group), melanoma on chronically sun damaged skin (64% of all/22.2% in the age group), and multicomponent asymmetric (26.4% of all/33.3% in the age group). Commonly present were features of regression under dermoscopy (54.1% of all/45.8% in the age group) and the co-occurrence of NMSC (64.29% of all/45.57% in the age group) (Figure 1).

### 3.3. Crude (Unadjusted) Odds Ratios for the Age Groups (Table 2)

The summary of the odds ratios, 95% confidence intervals, and *p*-value results for the clinical, dermoscopic, and epidemiologic characteristics of the melanoma patients in the individual age groups are presented in Table 2. 

**Figure 2:**
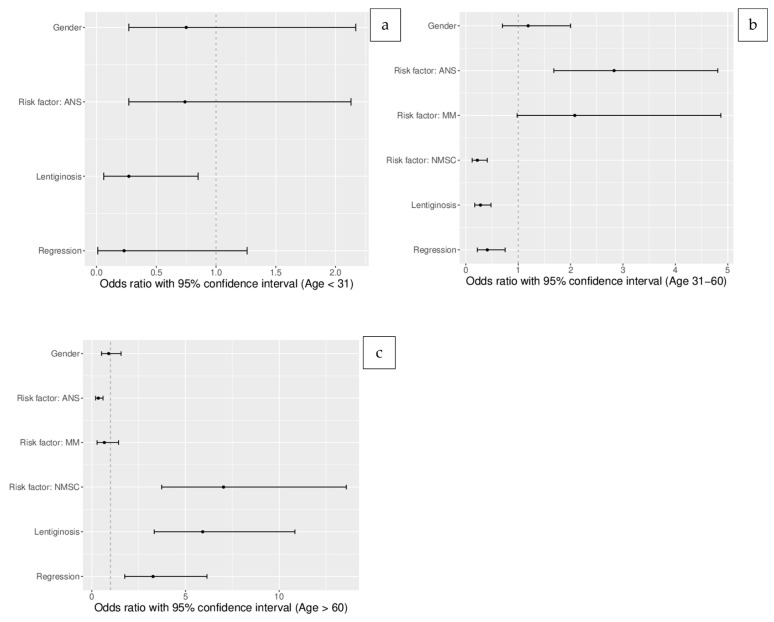
The characteristics of individual age groups of melanoma patients presenting unadjusted odds ratios with 95% confidence interval (*p* < 0.05 was considered statistically significant): (**a**) age group below 31 years old; (**b**) age group 31–60 years old; (**c**) age group over 60 years old.

The analysis of the group of patients younger than 31 years old revealed an absence of NMSC; thus, odds ratios were not calculated. In comparison to other age groups, there was a significantly lower prevalence of solar lentiginosis (OR 0.26, 95% CI: 0.04–1.06, *p* > 0.05). None of the other evaluated factors differed significantly from those of the other age groups (Figure 2a). 

In the group of patients aged 31–60 years, the statistical significance of odds difference was reached for ANS/NAN (OR 3.69, 95% CI: 1.99–6.99, *p* < 0.00001). The incidences of NMSC (OR 0.14, 95% CI 0.06–0.31, *p* < 0.000001), dermoscopic regression (OR 0.41, 95% CI: 0.22–0.75, *p*< 0.005), and solar lentiginosis (OR 0.22, 95% CI: 0.12–0.41, *p* < 0.000001) were lower than in other age groups (Figure 2b).

The statistical significance of incidences of multiple factors in the group of patients over 60 years old was revealed: previous/concomitant NMSC (OR 10.53, 95% CI: 4.81–24.94, *p* < 0.000001), the presence of solar lentiginosis (OR 7.27, 95% CI: 3.74–14.74, *p* < 0.000001), regression under dermoscopy (OR 3.27, 95% CI 1.76–6.15, *p*< 0.0001), and ANS/NAN (OR 0.24, 95% CI: 0.13–0.46, *p* < 0.00001) (Figure 2c).

The limitation of the study was the retrospective analysis and the low statistical power of the group of patients below 31 years old due to the small sample size. The analysis of melanoma risk factors was entirely based on empirical data collected from medical procedures.

## 4. Discussion

Morbidity from melanoma is increasing worldwide, especially in fair-skinned populations in all age groups [1,3]. Cutaneous melanoma mostly occurs in patients between 40 and 60 years old but is the most common form of cancer in young adults between 25 and 29 years old [1,7,12,13,14]. Currently, melanoma secondary prevention programs cover a small percentage of the adult population due to the lack of data supported or refuted by survival analyses from large randomized controlled trials, the high workloads of specialists, and the high economic costs [7,13,14,15]. As a result, they are mainly implemented via the opportunistic screening and surveillance of high-risk patients, as well as recommendations for self-examination and skin awareness to the general population [14]. The effectiveness of opportunistic screening was covered in studies by Argenziano et al. and Omara et al. [16,17]. 

Dermoscopy is one of the most important pillars of the secondary prevention of melanomas [18,19,20,21]. It enables their diagnosis at an early stage and thus contributes to a reduction in melanoma mortality [18,19,20,21]. Recently, Kalloniati et al., in a comparative study, showed 78.2% sensitivity and 71.4% specificity in identifying the clinical atypia based on naked eye examinations versus 89.1% sensitivity and 93.7% specificity based on dermoscopy [22]. Nazzaro et al. discussed the reasons for the increased incidence and diagnosis of thin melanomas (especially in situ and in small-sized melanomas) observed within the years 2006–2020 [23]. The authors pointed out the possible coexistence of two potential causes of this trend. The first resulted in the rapidly growing global incidence of melanoma, especially in white populations, and the second resulted in overdiagnosis. The latter could be derivative of the increasingly common use of dermoscopy, the use of new diagnostic methods (such as reflectance confocal microscopy), greater patient awareness, and healthcare-seeking behavior for skin cancer screening. In our opinion, all the above aspects should be taken into account, considering the fact that melanomas with a nodular component (spizoid and non-spitzoid) are characterized by a different histogenetic subtype, which was the subject of recent publications by Dessinioti et al., Raghavan et al., and Pusiol et al. [24,25,26]. BRAF mutations are more likely to be detected in SSM, while NM is associated more strongly with the ‘non-nevus’ melanoma pathway development model and NRAS mutations [24]. A SEER registry study reported that median Breslow thickness decreased for SSM over the period 1989–2009, whereas median Breslow thickness increased for nodular melanoma (NM) over time [27]. NM has been reported more frequently in older patients (>50 years), specifically in older men compared to SSM, and with low nevus counts [24]. Lower total-body nevus counts have been associated with thicker melanoma. These findings highlight a subset of melanoma patients that do not have well-established risk factors, i.e., high nevus count, and that could be at a higher risk for thicker aggressive melanomas [24]. The incidence of NM in our study was low (5.7%) and similarly frequent in the middle-aged and the oldest patients. Due to the small sample size of NM, no statistical correlation with the incidence of the risk factors could be performed. Although all the international melanoma screening guidelines stated the effectiveness of conducting skin examinations in patients harboring melanoma risk, specific recommendations on the age at which this screening should begin and the optimal visit intervals are lacking [1,7,14,28,29,30]. Moreover, no unique scoring system has been developed to discriminate high-risk versus low-risk individuals with respect to melanoma development [14]. As the elderly population presents the highest morbidity and mortality rates of melanoma, Nagore et al. investigated the impact of 17 risk factors for the development of melanoma in this population [4]. The authors found the strongest impact for fair eyes, severe sunburns, years of occupational sun exposure, smoking, over 50 melanocytic nevi, and a personal history of NMSC or other non-cutaneous neoplasms [5]. Tobacco smoking was an independent risk factor for cutaneous melanoma in this group.

Patients with melanoma are at increased risk of developing melanoma and NMSC, but also patients with NMSC have increased risk of melanoma and NMSC development [1,2,3,30,31,32,33]. Given the high global incidence of NMSCs, their comorbidity with melanomas is not that common. The overall prevalence of NMSCs was 7% among melanoma cases in the study by Neale et al. and 21.7% in our study [31]. In contrast, many melanoma patients never experience NMSCs, even BCCs, which share a similar pattern of UV exposure [14,32,33]. 

Our publication describes a population of adult patients both self-referring to a skin screening and those who were referred to this examination by other specialists who considered them to be at a high risk. The two sites participating in this study are both dermatological centers specialized in non-invasive skin cancer diagnostics providing dermoscopy, digital monitoring under videodermoscopy, and reflectance confocal microscopy (RCM) examinations in selected cases. The importance of secondary prevention in such a population might be proved by our findings: 84.7% of melanomas were preinvasive, 61.7% were detected during the first dermoscopic visit, and 38.3% were detected during the monitoring visits. Furthermore, 10% (19/189) of patients were diagnosed with more than one melanoma, among whom five patients presented with two concomitant melanomas during the first skin examination. In other patients, secondary melanomas were diagnosed between 6 months and 40 years following the primary diagnosis. In the context of educational campaigns encouraging periodic dermoscopic examinations, the repeated correlation of the higher frequency of early melanomas (including small melanomas) in middle-aged women is also noteworthy [7,13,24,34,35,36,37]. This is probably due to the fact that this population of patients is the most willing to come for screening [34]. Given the huge increase in the incidence of melanoma, it seems particularly important to direct the media’s message to groups of middle-aged men. Perhaps the delay in the diagnosis of this group of patients is the reason for the observed change in the proportions between the sexes in the group of middle-aged (predominance of women) and elderly (predominance of men or similar frequency for both sexes) patients [Supplement S2]; Refs. [1,13,24]. This might also be the reason for the more frequent diagnosis of advanced melanomas in elderly men [13,24,37].

The analysis of the three age groups of patients revealed differences in the presence of patient-related risk factors, topographical, and dermoscopic and histological aspects of detected melanomas. The incidence of melanoma in the youngest age group was rare (5.7% of our study population). What we found very interesting was that the statistical analysis excluded the correlation with common melanoma risk factors. Furthermore, the youngest group of adult patients (18–30 years) showed a dermoscopic similarity of melanomas with the patients described by Carrera et al. regarding the pediatric population—a spitzoid (pigmented or non-pigmented) and non-spitzoid (predominant multicomponent asymmetric) pattern [38]. Recently, De Giorgi et al. indicated a new finding—that pediatric melanomas were predominantly pigmented (95% of their cases) and characterized by at least two melanoma-specific structures in dermoscopy, among which the streaks and pseudopods (typical for the spitzoid pattern) were the most commonly found [39]. This could be explained by recent data, which indicated that genetic, rather than UV-induced, factors are responsible for the evolution of spitzoid lesions (Spitz/Reed nevi, spitzoid melanoma, and atypical spitzoid tumours) [25]. Based on the above data, younger populations should be educated to pay special attention to newly emerging and fast-growing lesions that are pink or dark brown and black in color and nodular in palpation, matching the features of spitzoid lesions [40,41,42]. In a study by Costa et al., 25.8% of those lesions were melanomas in the age group of 20–31 years [42]. Therefore, young patients with multiple growing nevi require close digital dermoscopy monitoring and the excision of Reed/Spitz-looking lesions [40,41,42]. The remaining frequently detected dermoscopic pattern in patients aged below 31 years was the multicomponent asymmetric one. It should be easily detected even based on the clinical ABCDE melanoma criteria, although with the risk of early invasive melanoma detection [43].

The incidence of patient-related risk factors showed their higher occurrence among patients between 31 and 60 years of age. In our study, this population was characterized by multiple acquired melanocytic nevi/ANS, the individual or familial occurrence of melanoma, the co-occurrence of NMSC, and solar lentiginosis. Moreover, we found the greatest clinicopathological and dermoscopic diversity of melanomas. All five cases of nevus-associated melanomas, most of the small-sized melanomas, and melanomas in special locations were found (although facial melanoma was more common in the older population). Summarizing the above results, the middle-aged population may require extensive dermoscopic examinations, precision in total-body skin examinations, the digital monitoring of selected lesions, and, perhaps, total-body photography and reflectance confocal microscopy (RCM) in order to reduce the number of unnecessary excisions and increase diagnostic accuracy.

The characteristics of melanoma patients aged over 60 years in our study confirmed earlier findings regarding the common presence of solar lentiginosis; comorbidity with NMSC; regression structures under dermoscopy; and the location of melanomas in the face, trunk, and upper limbs [4,37,44]. Contrary to the study by Nagore et al., the incidence of common risk factors—multiple melanocytic nevi/ANSs and a personal/family history of melanoma—were not statistically significant in our group. Generally, among those patients, we found two subgroups of melanomas. The first, with striking signs of melanomas, presented as facial or superficial spreading melanomas. The second—with great difficult in detection—presented as simulators of solar lentigines or regressed seborrheic keratoses, which present as light brown, pink, and grey in color. This is why the most common dermoscopic patterns found in this age group were the melanoma pattern on chronic sun-damaged skin (including extrafacial lentigo maligna), the multicomponent asymmetric pattern, and the homogenous pattern, which was consistent with previous observations [45,46]. Although many studies reported a higher incidence of nodular melanoma resulting in lower 10-year disease-specific survival among men over 65, we have not found a higher incidence of primary nodular melanomas (the advanced superficial spreading melanomas with a nodular component are not included in this category) in this age group [4,14,37,47,48]. Regarding the common detection of melanoma on the head in elderly patients, it is extremely important to focus on scalp melanomas (SM) due to their poor prognosis and difficult dermoscopic diagnostics. In a recent publication, Porto et al. showed that SM presents differently depending on the comorbidity with androgenetic alopecia and that patients without alopecia had higher Breslow thickness due to late diagnosis (because of hair concealment) [49]. Furthermore, SM has a distinct molecular profile with a high frequency of BRAF V600K, NF1 mutations, and a high frequency of detrimental mutations, which might explain their poor prognosis.

Our additional investigation aimed to describe the correlation between NMSC, as a melanoma risk factor, with the clinical and dermoscopic features of melanoma patients and showed higher odds ratios with statistical significance in the age group above 60 years and in patients with solar lentiginosis symptoms. Those findings have a practical impact as, by revealing the presence of NMSC and solar lentiginosis in elderly or middle-aged patients during skin examination, we might expect certain types of melanoma—characteristic for chronic sun-damaged skin—which should increase dermoscopists attention to regressed and difficult-to-find lesions.

The results of our study proved the necessity for closer surveillance of middle-aged patients, among whom melanomas are diagnosed most. These patients not only frequently harbor different risk factors but also may present a wide diversity of difficult-to-diagnose melanomas. Therefore, middle-aged patients will benefit to the greatest extent from the advantages of systematic diagnostic workflows, including RCM [21]. Lastly, this group also has a poor projected prognosis regarding mortality caused by melanoma in 2040 among patients over 60 years of age as those data describe the current middle-aged population [1].

## 5. Conclusions

The striking differences in the prevalence of common melanoma risk factors, melanoma-specific dermoscopic patterns, and topography were found among the three age groups.

Among the youngest and the oldest patients, no correlation with common melanoma risk factors was found. Patients aged 18–30 years present pediatric dermoscopic patterns of melanoma—the spitzoid and multicomponent asymmetric ones. The patients aged over 60 years presented consequences of the effects of sun exposure over a lifetime (both intermittent and cumulative solar damage or sunburns), which were represented by solar lentiginosis, comorbidity with NMSC, LM/LMM, and the SSM histological subtype, as well as the regression structures characteristic for the multicomponent asymmetric pattern and melanoma on sun-damaged skin dermoscopic patterns.

The incidence of patient-related risk factors showed a higher occurrence among patients between 31 and 60 years of age, which showed the highest general melanoma detection rate. Moreover, we found the greatest clinicopathological and dermoscopic diversity of melanomas (including the highest incidence of small-size melanomas). This makes the middle-aged group the most in need of precise examinations and digital monitoring.

Knowledge of age group differentiation factors may increase the precision of dermoscopic and dermatological surveillance and might influence the content and targeting of melanoma secondary prevention campaigns.

## Figures and Tables

**Table 1:** Epidemiological, clinical, histopathological, topographical, and dermoscopic data of patients diagnosed with melanoma divided into three age groups and calculated based on Fisher’s exact test for count data with simulated *p*-value (*p* < 0.05 was considered statistically significant). NS—not statistically significant.

	Total	Age < 31 y	Age 31–60 y	Age > 60 y	*p* Value
	n (%)	n (%)	n (%)	n (%)	
		Within the Group	Within the Group	Within the Group	
**Gender**		12	113	64	NS
Female	120 (63.5%)	6 (50.0%)	71 (62.8%)	43 (67.2%)	
Male	69 (36.5%)	6 (50.0%)	42 (37.2%)	21 (32.8%)	
**History of personal/familial melanoma**					NS
Yes	24 (12.5%)	0 (0%)	18 (15.9%)	6 (9.4%)	
No	165 (87.3%)	12 (100.00%)	95 (84.1%)	58 (90.6%)	
**Multiple acquired nevi/Atypical nevus syndrome**					<0.0005
Yes	121 (64.0%)	8 (66.7%)	86(76.1%)	27(42.2%)	
No	68 (36%)	4 (33.3%)	27 (23.9%)	37 (57.8%)	
**Skin phototype**					NS
I	6 (3.2%)	3 (25.0%)	2 (1.7%)	1 (1.5%)	
II	177 (93.6%)	9 (75.0%)	107 (94.7%)	61 (95.3%)	
III	6 (3.2%)	0 (0%)	4 (3.5%)	2 (3.1%)	
IV–V	0 (0%)	0 (0%)	0 (0%)	0 (0%)	
**Solar lentiginosis**					<0.0005
Yes	81 (42.9%)	2 (16.7%)	32 (28.3%)	47 (73.4%)	
No	108 (57.1%)	10 (83.3%)	81 (71.7%)	17 (26.6%)	
**Histopathological report:**					<0.01
- LM	25 (12.0%)	0 (0%)	10 (8.0%)	15 (20.8%)	
- LMM	4 (1.9%)	0 (0%)	0 (0%)	4 (5.5%)	
**Melanoma:**					
- pTis	47 (22.5%)	2 (16.6%)	28 (22.4%)	17 (23.6%)	
- pT1	101 (48.3%)	7 (58.3%)	69 (55.2.%)	25 (34.7%)	
- pT2	24 (11.5%)	2 (16.6%)	13 (10.4%)	9 (12.5%)	
- pT3	3 (1.4%)	1 (8.3%)	1 (0.8%)	1 (1.4%)	
- pT4	5 (2.4%)	0 (0%)	4(3.2%)	1 (1.4%)	
**Dermoscopic pattern of melanoma**					<0.0005
Multicomponent asymmetric	91 (43.5%)	4 (33.3%)	63 (50.4%)	24 (33.3%)	
Spitzoid	37 (17.7%)	6 (50.0%)	28 (22.4%)	3 (4.2%)	
Melanoma on sun damaged skin	25 (12.0%)	0 (0%)	9 (7.2%)	16 (22.2%)	
Hypomelanotic/amelanotic	10 (4.8%)	0 (0%)	7 (5.6%)	3 (4.2%)	
Homogenous	5 (2.4%)	0 (0%)	4 (3.2%)	1 (1.4%)	
Nodular	12 (5.7%)	1 (8.3%)	6 (4.8%)	5 (6.9%)	
Melanoma on face	22 (10.5%)	0 (0%)	3 (2.4%)	19 (26.4%)	
Melanoma in special location (nail apparatus/acral/mucous membranes)	7 (3.3%)	1 (8.3%)	5 (4.0%)	1 (1.4%)	
**Dermoscopic structures of regression**					<0.001
Yes	61 (29.2%)	1 (8.3%)	27 (21.6%)	33 (45.8%)	
No	148 (70.8%)	11 (91.7%)	98 (78.4%)	39 (54.2%)	
**Melanoma location:**					<0.0005
- Head and neck	25 (11.9%)	0 (0%)	6 (4.8%)	19 (26.%)	
- Trunk	70 (33.5%)	4 (33.3.%)	46 (36.8%)	20 (27.8%)	
- Upper limb	40 (19.1%)	3 (25.0%)	22 (17.6%)	15 (20.8%)	
- Lower limb	71 (34.0%)	4 (33.3%)	49 (39.2%)	18 (25.0%)	
- Nail apparatus	1 (0.5%)	1 (8.3%)	0 (0%)	0 (0%)	
- Mucous membrane	2 (1%)	0 (0%)	2 (1.6%)	0 (0%)	
**Previous/concomitant NMSC**					<0.0005
Yes	41 (21.7%)	0 (0%)	10 (8.9%)	31(48.4%)	
No	148 (78.3%)	12 (100%)	103 (91.1%)	33 (51.6%)	

**Table 2:** Summary of odds ratios, 95% confidence intervals, and *p*-value results for clinical, dermoscopic, and epidemiologic characteristics of melanoma patients in individual age groups. *p* < 0.05 was considered statistically significant; NS—not statistically significant; ND—no data.

Factor	OR	95%CI	*p*-Value
Characteristics of melanoma patients below 31 years of age (unadjusted)			
Gender (male)	1.8	0.53–6.15	NS
Melanoma (previous/concomitant or in family history)	ND	ND	ND
Atypical nevus syndrome or multiple acquired nevi	1.11	0.33–4.46	NS
NMSC (previous/concomitant)	ND	ND	ND
Regression under dermoscopy	0.23	0.01–1.26	NS
Solar lentiginosis	0.26	0.04–1.06	NS
Characteristics of melanoma patients between 31 and 60 years of age (unadjusted)			
Gender (male)	0.7	0.59–1.98	NS
Melanoma (previous/concomitant or in family history)	2.17	0.85–6.34	NS
Atypical nevus syndrome or multiple acquired nevi	3.69	1.99–6.99	<0.00001
NMSC (previous/concomitant)	0.14	0.06–0.31	<0.000001
Regression under dermoscopy	0.41	0.22–0.75	<0.005
Solar lentiginosis	0.22	0.12–0.41	<0.000001
Characteristics of melanoma patients 60 years old (unadjusted)			
Gender (male)	0.79	0.41–1.48	NS
Melanoma (previous/concomitant or in family history)	0.63	0.21–1.6	NS
Atypical nevus syndrome or multiple acquired nevi	0.24	0.13–0.46	0.00001
NMSC (previous/concomitant)	10.53	4.81–24.94	<0.000001
Regression under dermoscopy	3.27	1.76–6.15	<0.0001
Solar lentiginosis	7.27	3.74– 14.74	<0.000001

## Data Availability

The data presented in this study are available on request from the corresponding author. The data are not publicly available due to privacy and ethical restrictions.

## Supplementary Materials

The following supporting information can be downloaded at https://www.mdpi.com/article/10.3390/life13061369/s1. Supplement S1 [8]; Supplement S2 [9].
