# Supplementary material for: Clinical, Dermoscopic, and Histological Characteristics of Melanoma Patients According to the Age Groups: A Retrospective Observational Study
Source: Life (Basel). 2023 Jun 12;13(6):1369. doi: 10.3390/life13061369 (PMC10305549; doi:10.3390/life13061369)

Supplement S1 [8]

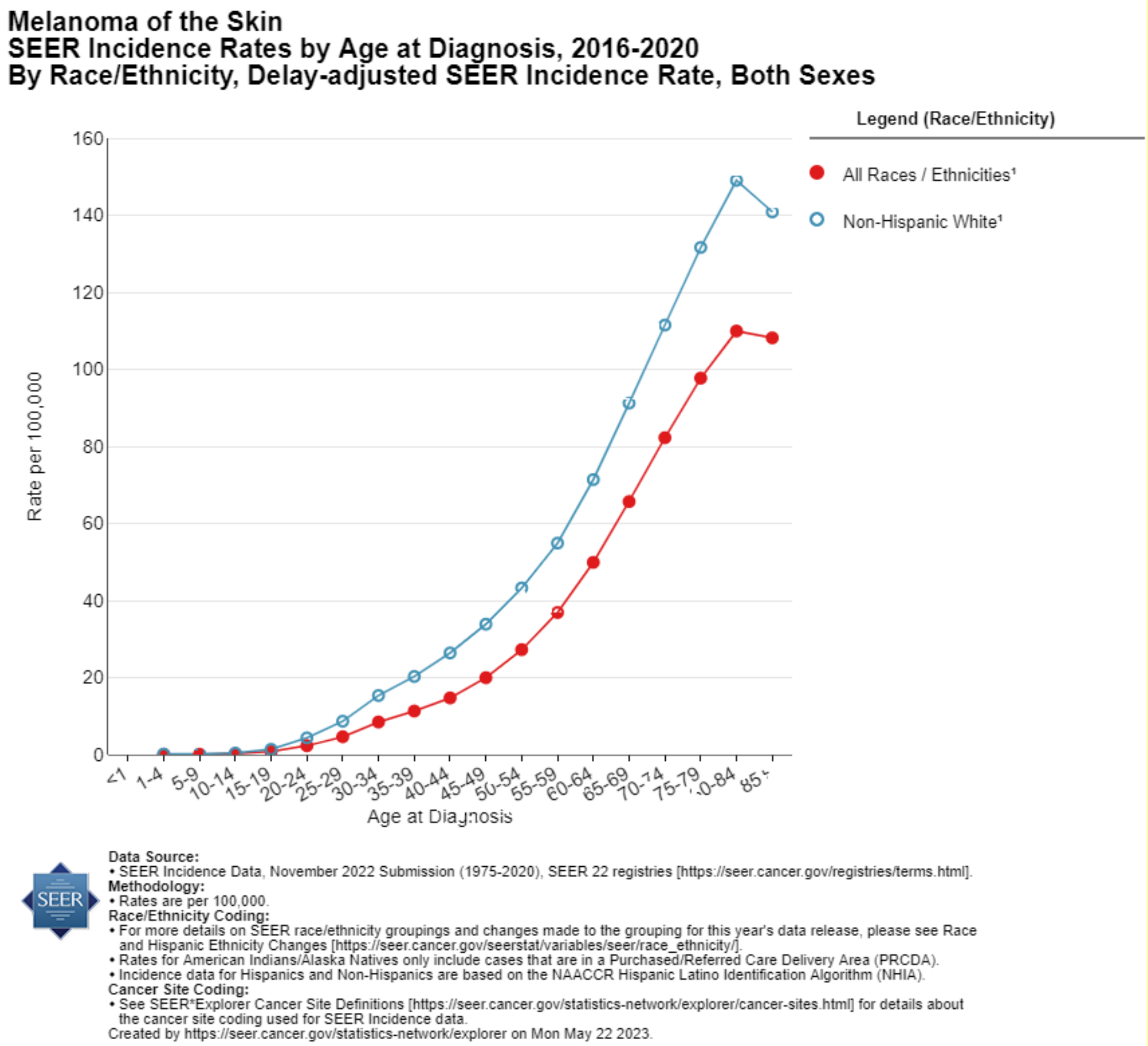

Supplement S2 [9]

Zachorowania/Zgony

Trendy czasowe

KRAJOWY REJESTR NOWOTWORÓW

Grupuj według Kod nowotworu, Grupa wiekowa
Metryki
Statystyka
Płeć
Nowotwory
Region
Grupy wiekowe
Przedział czasowy

Select according to Neoplasm code, Age groups
Metrics Incidence (Morbidity)
Statistics Crude ratio
Gender Males (purple), Females (green)
Neoplasm Melanoma
Age groups 0-89
Timeline 2016-2020

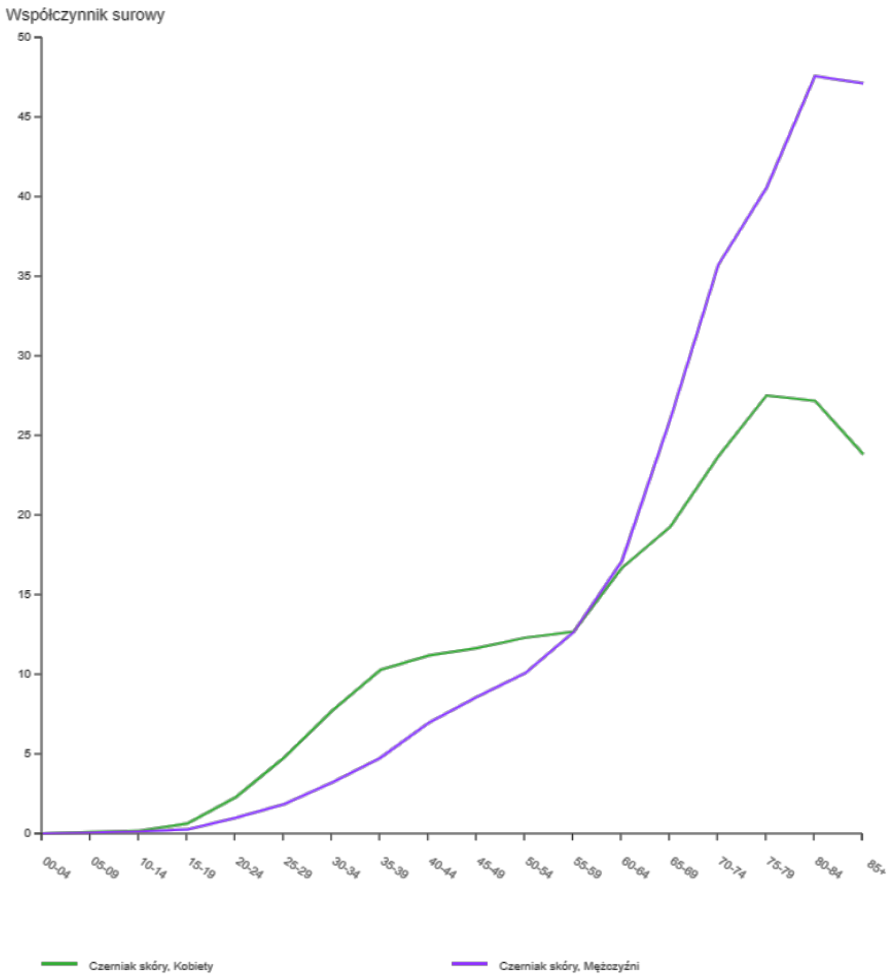

Supplement: Supplementary file 1 [file life-13-01369-s001.zip › life-2375901-supplementary.pdf]
